# Phosphoregulation on mitochondria: Integration of cell and organelle responses

**DOI:** 10.1111/cns.13141

**Published:** 2019-04-25

**Authors:** Maribel Lucero, Ana E. Suarez, Jeremy W. Chambers

**Affiliations:** ^1^ Department of Environmental Health Sciences, Robert Stempel College of Public Health & Social Work the Biomolecular Sciences Institute Florida International University Miami Florida

**Keywords:** A-kinase anchoring protien, c-Jun N-terminal Kinase (JNK), leucine-rich repeat kinase 2 (LRRK2), PTEN-induced kinase-1 (PINK-1) mitochondria, outer mitochondrial membrane, protein phosphatase, Sab (SH3-binding protein 5; SH3BP5)

## Abstract

Mitochondria are highly integrated organelles that are crucial to cell adaptation and mitigating adverse physiology. Recent studies demonstrate that fundamental signal transduction pathways incorporate mitochondrial substrates into their biological programs. Reversible phosphorylation is emerging as a useful mechanism to modulate mitochondrial function in accordance with cellular changes. Critical serine/threonine protein kinases, such as the c‐Jun N‐terminal kinase (JNK), protein kinase A (PKA), PTEN‐induced kinase‐1 (PINK1), and AMP‐dependent protein kinase (AMPK), readily translocate to the outer mitochondrial membrane (OMM), the interface of mitochondria‐cell communication. OMM protein kinases phosphorylate diverse mitochondrial substrates that have discrete effects on organelle dynamics, protein import, respiratory complex activity, antioxidant capacity, and apoptosis. OMM phosphorylation events can be tempered through the actions of local protein phosphatases, such as mitogen‐activated protein kinase phosphatase‐1 (MKP‐1) and protein phosphatase 2A (PP2A), to regulate the extent and duration of signaling. The central mediators of OMM signal transduction are the scaffold proteins because the relative abundance of these accessory proteins determines the magnitude and duration of a signaling event on the mitochondrial surface, which dictates the biological outcome of a local signal transduction pathway. The concentrations of scaffold proteins, such as A‐kinase anchoring proteins (AKAPs) and Sab (or SH3 binding protein 5—SH3BP5), have been shown to influence neuronal survival and vulnerability, respectively, in models of Parkinson's disease (PD), highlighting the importance of OMM signaling to health and disease. Despite recent progress, much remains to be discovered concerning the mechanisms of OMM signaling. Nonetheless, enhancing beneficial OMM signaling events and inhibiting detrimental protein‐protein interactions on the mitochondrial surface may represent highly selective approaches to restore mitochondrial health and homeostasis and mitigate organelle dysfunction in conditions such as PD.

## 1 BACKGROUND

**Figure 1:**
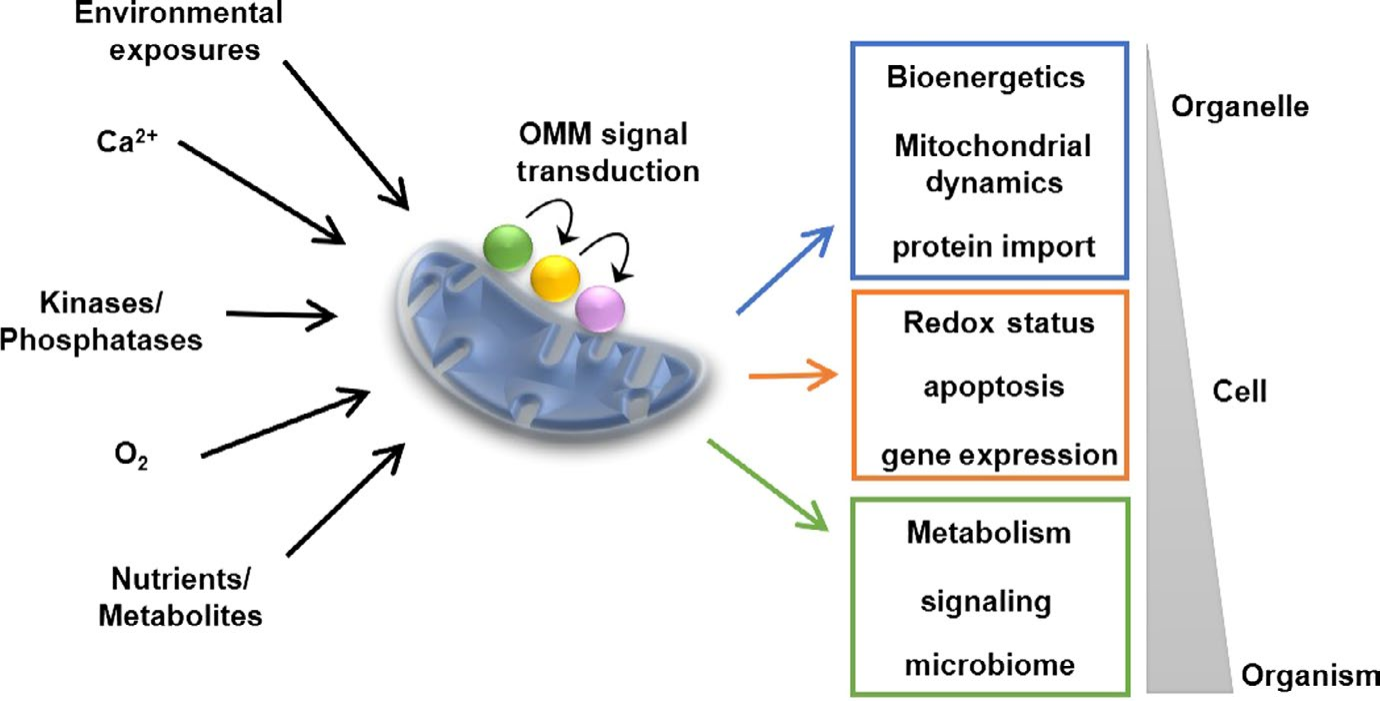
Mitochondrial signaling mechanisms regulate organelle, cell, and organisms physiology. Mitochondria are influenced by the intracellular and extracellular stimuli such as ions, metabolites, and molecules in the environment like oxygen and pesticides. However, the impact of signaling cascades in response to indirect actions of second messengers or stressors is emerging as significant manipulators of physiology. Mitochondrial signaling and second messengers have been shown to affect mitochondrial processes ranging from local events such as bioenergetics, mitochondrial dynamics, and proteins import to controlling transcriptional programs such as mitochondrial biogenesis, gene expression, and redox homeostasis. Of course, mitochondria are crucial to cell viability because the organelles are home to cell death machinery. Mitochondrial signaling can also transcend the cell through metabolites, second messengers, and even extracellular vesicles to impact metabolism, immune responses, inter‐tissue signaling, and the microbiota within an organism. Mitochondrial signaling has emerged as a critical component to human health and disease. The stimuli and transcriptional programs converge on signaling proteins on the outer mitochondrial membrane (OMM), which are uniquely positioned to receive and convey signals from both cell and organelle. Discrete OMM signaling events then coordinate mitochondrial and cellular responses to adapt organelle, cell, and organismal physiology to the current environment

Mitochondria are highly integrated organelles responsible for regulating cellular bioenergetics and viability in a continually changing environment. This extensive level of integration requires intricate and precise systems for communication with other cellular compartments. As the cellular environment changes, mitochondria must adjust their function, and cells must be able to detect, respond, and modulate mitochondria rapidly to adapt. Mitochondria have evolved sophisticated signaling mechanisms with cells that involve gases, ions, hormones, metabolites, and proteins.^1^ Thus, mitochondria are crucial hubs for receiving, integrating, and transmitting signals within cells.^2^ Environmental, cellular, and organelle‐based messengers impact all areas of mitochondrial physiology including genome integrity, bioenergetics, translation, and protein import (Figure 1).^3^

The quickness necessitated for accurately adjusting mitochondrial function to physiological perturbations implies that post‐translational modifications (PTMs) of local proteins are used before the induction of transcriptional programs. Reversible phosphorylation has emerged as a prominent mechanism for rapidly regulating mitochondrial protein function; in fact, recent proteomic studies indicate that ~40% of the organelle proteome is phosphorylated.^4^, ^5^, ^6^, ^7^, ^8^ Additionally, over 30 protein kinases and phosphatases are reported to migrate to mitochondria or have mitochondrial substrates.^6^, ^8^, ^9^, ^10^, ^11^ These phosphorylation events affect metabolism, mitochondrial dynamics, organelle function, and apoptosis demonstrating the importance of coordinated mitochondria‐cell communication.

The outer mitochondrial membrane (OMM) is the interface between mitochondria and the rest of the cell. Despite that most mitochondrial proteins reside within the inner mitochondrial membrane (IMM), the OMM hosts significant elements of mitochondrial physiology including organelle dynamics, protein import, and apoptosis. Consequently, protein kinases and phosphatases on the OMM are appropriately positioned to convey signals to and from mitochondria to directly manipulate mitochondrial form and function. The importance of OMM signaling is supported by numerous studies demonstrating that cytosolic kinases migrate to the organelle surface and modify local substrates to affect mitochondrial physiology.^8^, ^9^, ^11^ Neurons have a particular reliance on mitochondria for energy production, calcium buffering, and managing ionic changes related to synaptic transmission.^12^ Furthermore, dysfunction of OMM signaling components can contribute to the pathophysiology of neurodegenerative diseases, such as Alzheimer's disease (AD) and Parkinson's disease (PD).^9^, ^13^

In this review, we will identify the kinases and phosphatases found on the OMM (summarized in Table 1) of neurons and other CNS cells. We will present the molecular architecture required for each signaling protein and discuss the effects of specific substrate phosphorylation events including the impacts on organelle and neuronal physiology. We will highlight how perturbations in kinase and phosphatase activities can contribute to the pathophysiology of neurological diseases.

**Table 1:** Summary of select mitochondrial substrates of OMM kinases and phosphatases

Protein	Proposed substrates	Amino acid	Inhibit/activate	Pathway/activity	Suppress/promote	Identified on OMM	Ref.
Kinase							
*AMPK*	MFF AKAP‐1	Ser172 Ser103	Activate Activate	Fission Signaling	Promote Promote	Colocalization Phosphoproteomics	266 120
*Cdk1*	Drp‐1	Ser616	Activate	Fission	Promote	Colocalization	195
*Cdk5*	Drp‐1	Ser616	Activate	Fission	Promote	Colocalization	
*Cdk11*	Unknown			Apoptosis		Colocalization	203
*CKI*	Bid	Ser64/66	Inhibit	Apoptosis	Suppress	Colocalization	207
*CK2*	Bcl‐2 proteins FUNDC1 MIM1/2 TOM20 TOM22	Ser13 Ser12/16 Ser172 Ser44/46	Inhibit Inhibit Inhibit Inhibit Inhibit	Apoptosis Mitophagy Protein import Protein import Protein import	Suppress Suppress Suppress Suppress Suppress	Colocalization	207 210 123
*ERK1/2*	Bcl‐2 proteins Drp‐1 Mfn1 PGK‐1 StAR TRAP1	Ser616 Thr562 Ser203 Ser232 Ser511/568	Inh/Act Activate Inhibit Activate Activate Activate	Apoptosis Fission Fusion Metabolism Metabolism Metabolism	Supp/Prom Promote Suppress Suppress Promote Suppress	Colocalization	62 75 76 77 80 78
*JNK*	Bcl‐2 proteins Mfn2 PDH‐Eα1 SH3BP5 Smac/DIABLO smARF2	Ser27 TBD Ser421 Ser6 TBD	Inh/Act Inhibit Inhibit Activate Activate Activate	Apoptosis Fusion Metabolism Signaling Apoptosis Mitophagy	Supp/Prom Suppress Suppress Promote Promote Promote	Colocalization	34 57 52, 53 28 47 56
*LRRK2*	Bcl‐2 Miro MCU PRDX3	Thr56 TBD TBD Thr146	Activate Inhibit Activate Inhibit	Autophagy Trafficking Ca^2+^ Transit Antioxidant	Promote Suppress Promote Suppress	Colocalization	159 164 165 166
*mTOR*	Bcl‐xL	Ser62	Activate	Glycolysis	Promote	Colocalization	216
*PAK5*	Bad	Ser112	Inhibit	Survival	Suppress	Colocalization	223
*PINK1*	AKAP‐1 Parkin PINK1 Ub	TBD Ser65 Ser228/402 Ser65	Inhibit Activate Activate Activate	Signaling Mitophagy Mitophagy Mitophagy	Suppress Promote Promote Promote	Colocalization Proteomics	178 179 173 175
*PKA*	AIF1 Bad Bax Bim Drp1 GSTA4‐4 MIC19 MIC60 NDUFS4 TOM22 TOM40 TOM70 VDAC1	Ser39 Ser112/155 Ser60 Ser83 Ser637 Ser189 Thr11 Ser528 Ser173 Thr76 Ser54 Ser174 TBD	Inhibit Inhibit Inhibit Inhibit Inhibit Activate Inhibit Inhibit Activate Inhibit Inhibit Inhibit Inhibit	Bioenergetics Apoptosis Apoptosis Apoptosis Fission Antioxidant MICOS/Mitophagy MICOS/Mitophagy Bioenergetics Protein Import Protein Import Protein Import Ion Homeostasis	Promote Suppress Suppress Suppress Suppress Promote Suppress Suppress Promote Suppress Suppress Suppress Suppress	Colocalization Proteomics	131 95 129 130 121 105 105 110 124 125 126 267
*PKC*	Drp‐1 ECE‐1	Ser616 TBD	Activate Activate	Fission Amyloid Clearance	Promote Promote	Colocalization	149 152
*P38γ*	Bcl‐2 proteins Sab (SH3BP5)	TBD	Inh/Act Activate	Apoptosis Signaling	Supp/Prom Promote	Colocalization	81 86
Phosphatase							
*MKP‐1*	JNK MAPKs	Thr183/Tyr185	Inhibit Inhibit	Apoptosis ROS production	Suppress Suppress	Colocalization	224
*PP1*	AKAP1 Bad Bcl‐2 P38	TBD Ser112/136 Ser70 Thr180/Tyr182	Activate Activate Inhibit Inhibit	Signaling Apoptosis Apoptosis Signaling	Promote Promote Suppress Suppress	Colocalization	248 236 230 231
*PP2A*	Bad Bcl‐2 Drp1	Ser112 Ser70 Ser656	Activate Inhibit Activate	Apoptosis Apoptosis Fission	Promote Promote Promote	Colocalization Proteomics	238 237 95
*PTEN‐L*	Parkin Ubiquitin	Ser65 Ser65	Inhibit	Mitophagy	Suppress	Colocalization	229
*PTP1D*	AKAP1 Src	TBD Tyr530	Activate Activate	Signaling Bioenergetics	Promote Promote	Colocalization	239

## 2 OMM PROTEIN KINASES

Protein kinases influence mitochondrial protein function through phosphorylation. Intriguingly, most kinase activity within mitochondria can be attributed to tyrosine kinases such as Src.^14^, ^15^ Alternatively, much of the phosphorylation on the mitochondrial surface is associated with serine/threonine (Ser/Thr) protein kinases.^8^ Accordingly, the OMM protein kinases are almost exclusively Ser/Thr kinases, which we will discuss below.

## 3 MITOGEN‐ACTIVATED PROTEIN KINASES (MAPKS)

MAPKs, namely the c‐Jun N‐terminal kinase (JNK), extracellular regulated kinase (ERK1/2), and p38,^16^ exhibit transient mitochondrial localization in responses to cellular stimuli and stress. The principal targets of mitochondrial MAPKs appear to be the Bcl‐2 protein superfamily members, which explains the impact mitochondrial MAPKs have on the regulation of cell death pathways.^16^ Recent studies show that JNK, ERK, and p38 can phosphorylate OMM substrates involved in mitochondrial dynamics and bioenergetics.

### 3.1 c‐Jun N‐terminal kinase

The most well‐characterized MAPK with OMM localization is the c‐Jun N‐terminal kinase (JNK). There are three JNK isoforms (JNK1, JNK2, and JNK3) that are expressed in the brain, and studies from mice suggest that JNK isoforms account for most of the proline‐directed phosphorylation in the brain.^17^, ^18^ The activities of JNKs have been delineated in rodent brains with JNK1 accounting for most of the signaling in the cortex and cerebellum, and JNK3 accounts for the bulk of activity in the hippocampus and striatum.^19^, ^20^ Discrete subcellular may contribute to the diverse JNK substrates and physiological roles of JNK signaling in the brain with nuclear and cytosolic isoforms targeting different substrate pools. The cytosolic JNK species probably become the mitochondrial JNK species based on localization. Also, the functional redundancy and compensatory activities of JNK isoforms indicate that multiple JNKs could contribute to mitochondrial JNK signaling.^18^, ^21^, ^22^ In cerebral ischemia, researchers propose that JNK1 may be an early mitochondrial effector with JNK3 migrating to mitochondria later and inducing apoptosis.^23^ Similarly, in 1‐methyl‐4‐phenyl‐1,2,3,6‐tetrahydropyridine (MPTP)‐induced dopaminergic (DA) neuron degeneration in mice, ablation of both JNK2 and JNK3 was necessary to protect DA neurons from the neurotoxic insult.^24^, ^25^ These data imply that JNK1 is involved in physiological JNK signaling, while JNK2 and JNK3 are responsible for JNK‐mediated apoptosis; meanwhile, these studies highlight the potential for all JNK isoforms to localize on mitochondria.

Mitochondrial JNK signaling is linked to apoptosis in numerous tissues, including the brain, following cytotoxic stress. While the molecular mechanism of JNK translocation to the OMM remains to be determined, the scaffold protein, Sab (or SH3BP5), anchors JNK on mitochondria.^26^, ^27^, ^28^ Sab is a single‐pass transmembrane protein oriented in the OMM with the N terminus of the protein in the mitochondrial intermembrane space and the C terminus on the cytosolic face of the OMM.^29^ JNK interacts with Sab through kinase interacting motifs (KIM1/2) near the C terminus of Sab,^26^, ^28^, ^30^ and ablation of these sites or use of a competitive peptide (Tat‐Sab_KIM1_) can prevent JNK translocation to mitochondria.^26^, ^31^, ^32^ Disrupting the interaction between JNK and Sab is neuroprotective in rodent models of PD and cerebral ischemia.^23^, ^33^

**Figure 2:**
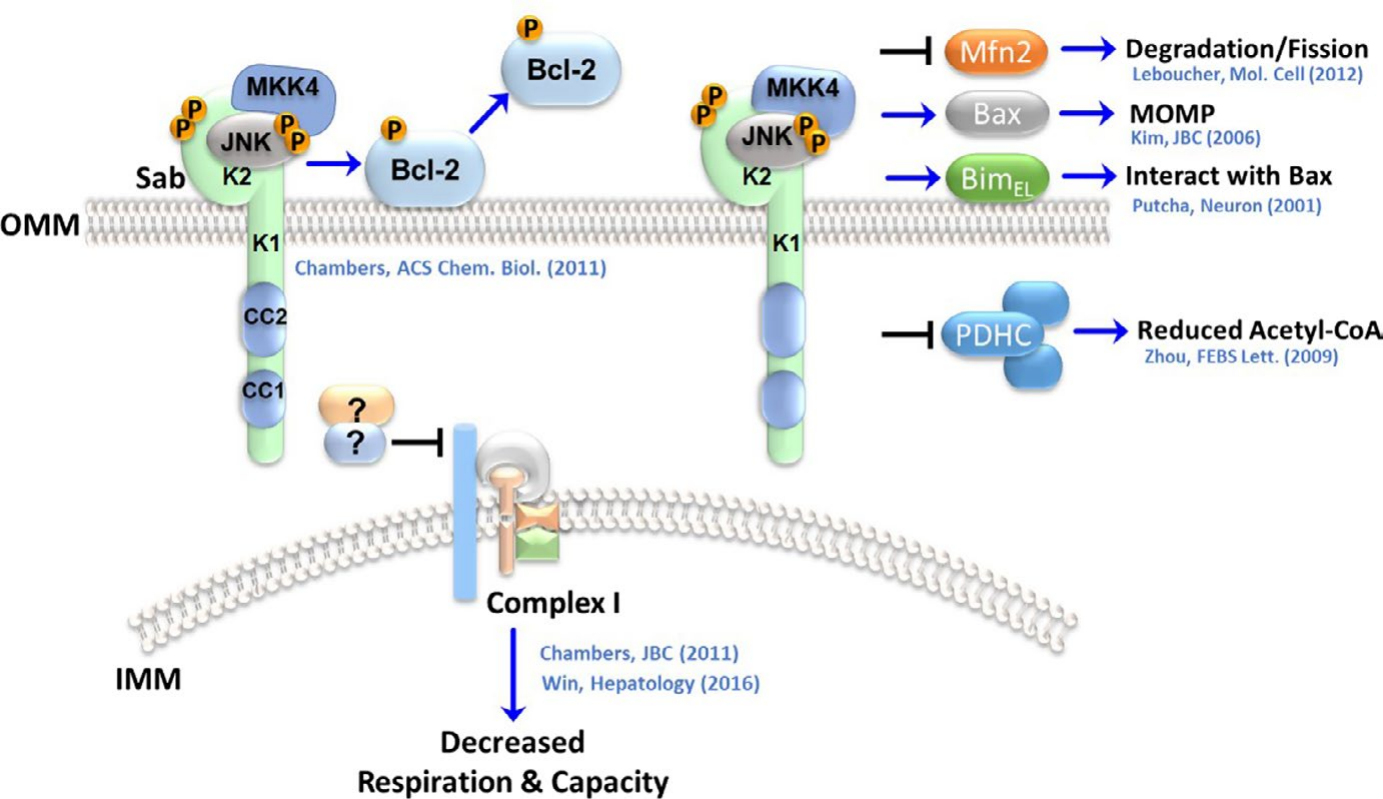
c‐Jun N‐terminal kinase (JNK) on the OMM impacts apoptosis and bioenergetics. JNK signaling has long been linked to cell death and metabolism, and only recently was it realized that mitochondrial JNK signaling on the OMM scaffold protein Sab was critical to the induction of apoptosis through the manipulation of proteins within the Bcl‐2 superfamily. Following activation, JNK translocates to mitochondria. JNK interacts with and phosphorylates the OMM scaffold protein Sab most likely through an interaction with the KIM2 motif (K2) because the KIM1 motif (K1) may be on the inner leaf of the OMM. In cases of neurotoxin exposure, ischemia, and cytotoxic stress, mitochondrial JNK activity leads to amplification of ROS production, organelle dysfunction, and cell death. Mitochondrial JNK can impair complex I by a yet‐to‐be‐described mechanism to impair mitochondrial metabolism and engage ROS production. However, this may be facilitated by signaling on the coiled‐coil motifs (CC1/CC2) of Sab's SH3 domain. Mitochondrial JNK can initiate apoptosis through phosphorylation of Bcl‐2 on Ser70 inducing its emigration from mitochondria. JNK activity on mitochondria has also been demonstrated to impact mitochondrial dynamics through phosphorylation of Mfn2, which leads to its degradation and mitochondrial fission. JNK also influences mitochondrial metabolism directly through the inhibition of PDH via phosphorylation of the Eα1 subunit. In addition to Bcl‐2, JNK can also influence the activities of BH3‐only proteins such as Bim to induce apoptosis, and mitochondrial JNK has been shown to phosphorylate Bax to induce permeabilization of the OMM. The local activities of mitochondrial JNK suggest it could be a significant physiological player in organelle health

Mitochondrial JNK activity impacts apoptosis, metabolism, and organelle dynamics (Figure 2); perhaps, the most widely identified JNK mitochondrial substrates are proteins from the Bcl‐2 superfamily.^34^ JNK phosphorylates many members of the Bcl‐2 superfamily to modulate their functions in stress, tissue, and cell‐dependent fashion. Concerning Bcl‐2 proteins in the CNS, JNK directly modifies Bcl‐2, Bad, and Bim and induces expression of apoptotic proteins, like activator of apoptosis Harakiri (HRK) to promote cell death in response to neurotoxic stress.^35^, ^36^, ^37^ Our research demonstrated that mitochondrial JNK could phosphorylate Bcl‐2 on Ser70 to induce emigration of Bcl‐2 from the OMM, in response to 6‐hydroxydopamine exposure in vitro and in vivo.^26^, ^33^ The loss of Bcl‐2 from the mitochondrial surface releases BH3‐only proteins and Bax to engage in mitochondrial outer membrane permeabilization (MOMP) initiating apoptosis.^38^, ^39^ Similarly, inhibition of pro‐survival OMM‐resident Mcl‐1 by JNK on Thr163 and Ser121 has been shown to promote apoptosis.^40^, ^41^ The inactivation of Mcl‐1 by JNK is an event implicated in neurotoxic degenerative models^42^ as well as elevated JNK activity in postmortem neurodegenerative brain tissue^43^, ^44^ reveals the importance of mitochondrial JNK inhibition of pro‐survival proteins in CNS disorders.

Alternatively, JNK has been shown to phosphorylate Bim on Ser65 during trophic factor deprivation (TFD) as part of apoptosis initiation. Again, JNK phosphorylation of Bim facilitated MOMP in Bax‐dependent apoptosis.^35^, ^37^ Other BH3‐only proteins have been documented as JNK substrates, including Bad, Bid, and Noxa.^34^ JNK directly phosphorylates Bax on Thr167 to promote oligomerization and pore formation in the OMM leading to the release of apoptotic contents of the intermembrane space.^45^ Smac/DIABLO, one of these intermembrane components, is a potential JNK substrate. Putative JNK phosphorylation of Smac/DIABLO mediates the release from mitochondria to ubiquitinylate inhibitors of apoptosis (IAPs) for degradation and sustains apoptosis.^46^, ^47^, ^48^ In addition to modifying mitochondrial resident Bcl‐2 superfamily proteins, JNK acts perimitochondrially in the cytoplasm to induce translocation of proteins such as Bad and Bax to mitochondria. This is achieved either by directly phosphorylating the proteins or by phosphorylation of their cytosolic scaffold, 14‐3‐3, to induce their release.^49^, ^50^, ^51^ Consequently, local JNK signaling on the OMM is a crucial component of neuronal apoptosis.

In addition to regulating cell viability, JNK signaling is associated with bioenergetics in the brain. The first evidence of the regulation of mitochondrial metabolism by local JNK signaling came from studies in the aging mouse brain.^52^, ^53^ JNK was found to phosphorylate pyruvate dehydrogenase (PDH) complex on the E1α subunit and impaired PDH activity.^52^, ^53^ JNK‐mediated inhibition of PDH leads to increase lactic acid levels and perhaps contributing to energetic deficits in the aging brain. Our research and studies in the liver by Win and colleagues 26, 29, 54, 55 demonstrate that JNK can impair the activity of respiratory complex I. Although the precise mechanism has yet to be determined, JNK translocation to the OMM is linked to enhanced ROS production. The direct inhibition of both PDH and complex I by JNK is problematic given that the substrates are within the mitochondria, and JNK had been shown to be present on the mitochondrial surface. Therefore, JNK phosphorylates the substrates either before or during protein import, unless indirect mechanisms may be responsible for the inhibition. This appears to be the case for complex I, as Win and associates found that inhibitors of intramitochondrial Src associate with Sab.^29^ Upon JNK binding/phosphorylation of Sab, Src inhibitor SHP‐1, a tyrosine phosphatase, is responsible for dephosphorylating Src Tyr419 inactivating the kinase and reducing complex I activity.^29^ These studies implicate mitochondrial JNK as a crucial metabolic regulator and illustrate the possibility of transmembrane signaling cascades at mitochondria, which may serve as intra‐ and extra‐organelle communication circuits.

Recently, JNK signaling has been linked to mitochondrial dynamics and quality control through the phosphorylation of OMM substrates,^56^, ^57^ and although this work was not performed in the CNS, the implications of impaired mitophagy and JNK signaling in neurodegenerative disorders warrant the mention of this relationship.^24^, ^43^, ^58^, ^59^ Specifically, JNK2 was found to translocate to mitochondria and phosphorylate the mitochondrial form of the alternate reading frame tumor suppressor [ARF (smARF, small ARF)] leading to ubiquitination and degradation by the proteasome.^56^ Diminished smARF levels raised the steady‐state levels of p62, a mediator of autophagic‐lysosomal degradation, which was more readily degraded in the absence of JNK2.^56^ Ablation of *JNK2* produced higher levels of basal mitophagy and autophagy and was typified by high smARF and low p62 (a result of increased autophagy) levels.^56^ Mitochondrial JNK activity has also been linked to the turnover of mitofusin‐2 (Mfn2) but not Mfn1. JNK was shown to phosphorylate Mfn2 on Ser27, which promoted the ubiquitination and proteasomal degradation of Mfn2.^57^ The loss of Mfn2 contributed to mitochondrial fragmentation in human U2OS osteosarcoma cells.

Additionally, the loss of Mfn2 was associated with induction of apoptosis to genotoxic stress induced by doxorubicin.^57^ Therefore, it is possible that local JNK signaling can contribute to the turnover of stressed mitochondria by enhancing fission and degradation of problematic organelles. Collectively, mitochondrial JNK is a crucial regulator of mitochondrial form and function as well as cellular viability in the CNS.

### 3.2 Extracellular regulated kinase

The extracellular regulated kinase (ERK) exists as numerous isoforms in the brain with ERK1/2 being the most well‐characterized species of the family. Similar to JNK, ERK1/2 activity on the mitochondria has been reported in the hippocampus,^60^ implicated in PD,^61^ and is associated with the post‐translational modification of Bcl‐2 family members 62 and organelle physiology.^63^, ^64^ Although ERK1/2 activity generally is thought to be pro‐survival, prolonged ERK1/2 signaling can be linked to cell death as well.^62^ ERK1/2 signaling outcomes are stimulus‐ and cell‐type‐dependent, as is the case with most MAPKs. Recently, ERK1/2 interaction with heat shock protein B1 (HSPB1) was shown to facilitate the phosphorylation of the BH3‐only protein Bim leading to its degradation and ultimately impairing ER stress‐induced apoptosis.^65^ Intriguingly, HSPB1 mutations from Charcot‐Marie‐Tooth disease exhibit high levels of BIM and are more vulnerable to ER stress‐induced cell death than their wild‐type counterparts.^65^ However, ERK1/2 phosphorylation of Bcl‐2 and Mcl‐1 has been described to have conflicting effects in the literature.^66^ For example, ERK1/2 phosphorylation of Bcl‐2 can prevent Bcl‐2 function activating neuronal apoptosis 67, 68, 69, 70; meanwhile, other reports indicate that ERK1/2 phosphorylation of Bcl‐2 promotes the protein's anti‐apoptotic activities.^71^, ^72^, ^73^ Similar studies have been noted for Mcl‐1 with ERK1/2 phosphorylation both inhibiting and enhancing Mcl‐1 anti‐apoptotic functions.^74^ Thus, considerable attention should be paid to the cellular and stress contexts of ERK1/2 signaling when examining Bcl‐2 phosphorylation especially in the diverse cellular populations of the CNS.

ERK1/2 activity has also been implicated as a regulator of mitochondrial dynamics. Mitochondrial ERK2 translocation emulates 6‐OHDA‐mediated effects on mitophagy.^61^ Additionally, mitochondrially localized ERK1/2 can phosphorylate dynamin‐related protein 1 (Drp‐1) and Mfn1 to impair fusion. It was reported in 2015 by two independent studies that ERK2 could phosphorylate Drp‐1 on Ser616 and promote mitochondrial fission.^75^ This event was shown to be driven by the Ras oncogene and necessary for tumor growth. ERK2‐Drp‐1 activation was later revealed to be required for cellular programming during development.^75^ Also, in 2015, Pyakural and colleagues demonstrated that ERK1/2 phosphorylation of Mfn1 on Thr562 impaired the organelle docking activities of Mfn1.^76^ Expression of constitutively active MAPK/ERK kinase (MEK) resulted in mitochondrial fragmentation. Also, ERK1/2 phosphorylation of Mfn1 also sensitized cells to apoptotic stimuli implicating mitochondrial ERK signaling in both functional and cell death responses.^76^

ERK1/2 signaling can impact bioenergetics and mitochondrial metabolism as well. Recent reports have suggested that ERK1/2 is necessary to induce glycolysis. One proposed mechanism for ERK regulation of glycolysis involves the phosphorylation of phosphoglycerate kinase 1 (PGK‐1) by ERK1/2 on Ser 203.^77^ This phosphorylation event induces mitochondrial PGK‐1 to become a protein kinase that phosphorylates and activates PDH kinase 1 (PDHK1) on Thr338. PDHK1, in turn, phosphorylates PDH inhibiting enzyme activity and promoting glycolysis.^77^ Another recent report demonstrates that in the presence of mutations in neurofibromin, a hallmark of neurofibromatosis type 1 (a condition that predisposes patients to tumor formation), ERK1/2 phosphorylates the chaperone TRAP1.^78^ Phosphorylation of TRAP1 impairs succinate dehydrogenase (SDH) leading to the accumulation of succinate and tumor progression.^78^ The most widely recognized metabolic interaction is between ERK1/2 and the steroidogenic acute regulatory protein (StAR).^79^ ERK1/2 phosphorylates StAR on Ser 232, which stabilizes the protein in mitochondria and sustains steroid synthesis. ERK activity, along with protein kinase A (PKA), is required for maximal steroid production in Leydig cells.^80^ These studies collectively implicate local ERK signaling in the regulation of mitochondrial form and function.

### 3.3 P38 kinases

The p38 kinases, in particular p38α (MAPK14), have been implicated in the pathogenesis of neurodegenerative, and pharmacological inhibitors of p38 have been shown to protect against neuronal loss in preclinical models of neurodegenerative disorders in the CNS.^81^, ^82^ The basis for these studies stems from the involvement of p38 in apoptosis. The general effect of p38 signaling affects the levels of Bcl‐2 (decrease) and Bax (increase) levels on mitochondria through transcriptional and post‐transcriptional means contributing to potentiating cells toward apoptosis.^83^ However, p38 direct effects on Bcl‐2 superfamily proteins remain elusive. Nonetheless, p38 has been shown to migrate to mitochondria follow stress (such as ischemia or oxidative stress) in cardiac tissue.^84^ Court and colleagues proposed that p38γ interacts with outer mitochondrial protein 25 (OMP25) that impaired p38γ activity in vitro.^85^ To date, mitochondrial p38, specifically p38γ, has been shown to converge on mitochondria and phosphorylate Sab, a well‐established mitochondrial scaffold protein for JNK.^86^ Investigators demonstrated in vitro p38γ phosphorylated Sab on Ser321,^86^ which is in KIM1, preceding a transmembrane motif.^29^ This places Ser321 within the mitochondria, and no documentation of p38 within mitochondria has been made raising questions to the contributions of this PTM to physiological mitochondrial signaling. Regardless, p38 is a significant player in signaling regulating mitochondrial health.

## 4 PROTEIN KINASE A, G, C (AGC KINASES)

The AGC kinase family has two members present on the OMM that can influence organelle physiology: PKA and isoforms of PKC.^87^ PKA is likely the most well‐investigated cytosolic kinase on the mitochondrial surface, as it is well documented to play a role in sustaining organelle health.^88^ Meanwhile, the role of PKC on mitochondria is more complicated with distinct variants contributing to the divergent aspects and outcomes of mitochondrial function and health.^89^, ^90^

### 4.1 Protein kinase A (PKA)

**Figure 3:**
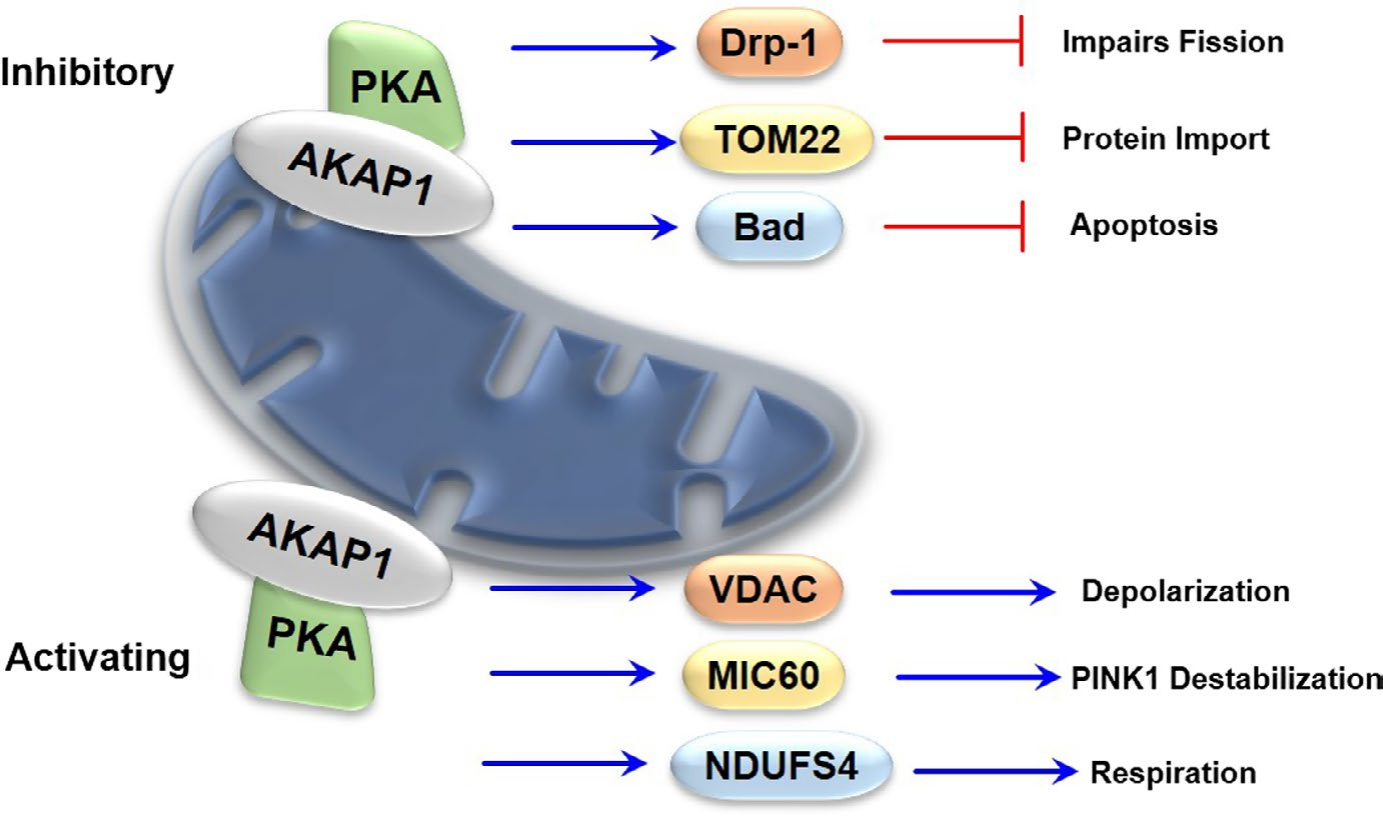
Mitochondrial PKA is a central mediator of mitochondrial health. PKA is a well‐documented regulator of mitochondrial form and function. The actions of PKA can be inhibitory to local processes or activate others. PKA interacts with AKAP1 (D‐AKAP) on the mitochondrial surface. PKA OMM activity can phosphorylate and impair the activity of Drp‐1 preventing fission and resulting in elongating mitochondria. Similarly, mitochondrial PKA activation of MIC60 can influence PINK1 stability on the OMM. Phosphorylation of MIC60 leads to the decline in PINK1 levels and prevent Parkin recruitment. This could be a potential mechanism for PKA to protect healthy mitochondria. Mitochondrial PKA can phosphorylate and inhibit the apoptotic actions of Bad. Alternatively, PKA can increase the function of mitochondrial proteins. Mitochondrial PKA can also phosphorylate VDAC and contribute to stress‐induced mitochondrial depolarization. Alternatively, PKA phosphorylation of OMM transporters such as TOM22 facilitates the import of nuclear‐encoded proteins (IE, subunits of the respiratory chain). PKA phosphorylation can coincide with protein import, as in the case of NDUFS4, a complex I subunit can enhance protein function and the efficiency of ETC complexes. These studies indicate mitochondrial PKA is required for healthy mitochondria

Mitochondrial PKA has long been associated with mitochondria through its interaction with scaffold proteins of the A‐kinase anchoring proteins (AKAP) family in particularly AKAP‐1 (or D‐AKAP as often used for the mitochondrial PKA scaffold).^91^, ^92^ PKA has been documented to affect numerous mitochondrial proteins ranging from bioenergetics to dynamics to redox homeostasis and cell survival.^93^ The substrates for mitochondrial PKA have been identified both on and within mitochondria suggesting the ability of mitochondrial PKA to transcend the OMM. Mitochondrial PKA signaling is thought to be mainly beneficial to the organelle facilitating essential functions such as protein import while impeding processes such as fission and apoptosis.^94^ Increasing the levels of mitochondrial PKA through genetic and pharmacological manipulation has been shown to be neuroprotective in preclinical models of neurodegenerative diseases, specifically PD.^95^, ^96^, ^97^ Furthermore, adding an OMM localization signaling to the N terminus of PKA can diminish the effects of mitochondrial toxins and stress.^95^, ^96^ These protective effects are best represented by the mitochondrial substrates of PKA on the OMM described immediately below and in Figure 3.

The most recent analysis of PKA with concerning OMM signaling and organelle physiology have involved the molecular machinery of mitochondrial dynamics.^98^ Mitochondrial PKA was first linked to organelle dynamics through phosphorylation of Drp‐1.^99^ PKA phosphorylation of Drp‐1 on Ser637 impairs the function of Drp‐1 preventing fission, which results in elongated mitochondria. Ablation of Drp‐1 Ser637 or dephosphorylation by calcineurin was found to activate Drp‐1 leading to organelle fragmentation.^99^ Increasing mitochondrial PKA was found to also protect neurons from neurotoxin‐induced cell death in part through phosphorylation of Drp‐1.^95^ The manipulation of Drp‐1 by PKA could be crucial to maintaining mitochondrial integrity and health and may affect mitochondrial transport in neurons as well as mitochondrial integration at synapses.^96^, ^100^, ^101^, ^102^, ^103^

PKA activity on the OMM has been shown to influence the stability of PTEN‐induced kinase‐1 (PINK1),^104^ which is a crucial component of mitophagy, through the phosphorylation of proteins in the mitochondrial contact site and cristae organizing system (MICOS).^105^ Increasing PKA mitochondrial activity was found to prevent Parkin recruitment to depolarized mitochondria by decreasing PINK1 levels. Investigators determined that PKA phosphorylation of MIC60, a MICOS component, on Ser528 destabilized PINK1 in the OMM by an unknown mechanism leading to its degradation.^105^ Meanwhile, mitochondrial PKA phosphorylation of MIC19 on Thr11 was found to impair Parkin recruitment to mitochondria. Also, elevating the levels of PKA on mitochondria reduced the amount of organelle depolarization in cultures.^105^ While these results indicate that PKA is involved in the regulation of mitophagy, it is exciting to think that coregulation of energetic and communication components of in MICOS can be manipulated by signal transduction pathways to influence organelle turnover and health. Perhaps, this interplay may represent a new therapeutic avenue for the treatment of neurodegenerative disorders, such as PD.^97^, ^104^

Mitochondrial PKA has been long associated with respiratory function and efficiency of complexes in the ETC through the manipulation of protein import and the direct phosphorylation of protein subunits of respiratory enzyme systems.^106^, ^107^, ^108^, ^109^ Mitochondrial PKA has been shown to directly affect the functions of ETC complexes I, IV, and V to promote respiration and ATP synthesis.^106^, ^107^, ^108^ A complex I target of PKA is NADH:ubiquinone oxidoreductase subunit S4 (NDUFS4). PKA phosphorylation of NDUFS4 on Ser173 promotes interaction with the Hsp70 chaperone facilitating NDUFS4 import into mitochondria and integration with other complex I subunits.^110^ Consequently, mutations that disrupt the NDUFS4 Ser173 phosphorylation site are linked to complex I dysfunction in the mitochondrial encephalomyopathy Leigh syndrome, a neuromuscular disorder associated with impaired mitochondria function.^111^, ^112^ In addition to modification of NDUFS4, mitochondrial PKA also influences the protein stability of complex I by inhibiting intramitochondrial proteases capable of degrading complex I constituents, NDUFA9, NDUFS4, and NDUFV2.^113^

Pharmacological inhibition of mitochondrial soluble adenylyl cyclase (sAC) results in the proteolytic degradation of these components due to diminished local PKA activity.^113^ Alternatively, complex IV (cytochrome c oxidase) subunits can be directly phosphorylated by mitochondrial PKA, and distinct modifications can serve to modulate complex function.^114^ For example, PKA phosphorylation of complex IV subunit I on Ser58 nullifies ATP‐dependent inhibition of complex IV sustaining activity, whereas PKA phosphorylation of subunits I, IV‐1, and Vb during hypoxia triggers degradation of the proteins and diminishes complex IV activity.^115^, ^116^

Similarly, mitochondrial PKA can modulate complex V activity. Local PKA phosphorylation of ATPase inhibitory factor 1 (AIF1) on Ser39 prevents the interaction between AIF1 and complex V maintaining ATP synthase activity.^117^, ^118^ Also, decreasing PKA activity in mitochondria can lead to the proteolytic degradation of complex V components resulting in less ATP synthesis.^118^, ^119^ The proximity of PKA to the bioenergetic machinery makes the kinase a useful regulator of ATP production and the ETC Furthermore, the relationship between ATP production and mitochondrial PKA activity extends to signaling transduction pathways. Wherein, activation of the AMP‐dependent protein kinase (AMPK) can phosphorylate and stabilize D‐AKAP to engage ATP production and promote cell survival.^120^ Mitochondrial PKA can be considered to be a crucial component of ETC and mitochondrial bioenergetic regulation.

Mitochondrial PKA is an embedded regulator of mitochondrial antioxidant capacity. Mitochondrial PKA can phosphorylate a glutathione S‐transferase variant (GSTA4‐4) on Ser189.^121^ Similar to NDUFS4, PKA phosphorylation of GSTA4‐4 facilitates its interaction with Hsp70 and subsequent import into mitochondria.^121^ This event enhances glutathione production in mitochondria and increases antioxidant capacity within organelles.

While mitochondrial PKA activity can increase the import of proteins by promoting interactions with chaperones like Hsp70, local PKA activity can phosphorylate protein transporters on the mitochondrial surface and impede their functions.^122^, ^123^ PKA phosphorylation of TOM22 on Thr76^124^ and TOM40 on Ser54^125^ can prevent transport of the proteins into mitochondria; additionally, the PKA phosphorylation of TOM22 can also impair its transport functions.^124^ Similarly, PKA can phosphorylate TOM70 on Ser174, which inhibits TOM70 receptor‐type activities toward chaperone functions such as Hsp70.^126^ To reconcile the distinctions between PKA's facilitation and inhibition of protein import into mitochondria, the levels, and duration as well as the context of PKA, as well as other signaling events, should be taken into account when observing mitochondrial outcomes.

Finally, PKA signaling on mitochondria was found to impair apoptosis.^127^, ^128^, ^129^, ^130^ Mitochondrial PKA can phosphorylate Bad on Ser112, and Ser155 has been shown to alleviate the inhibitory effects of Bad on anti‐apoptotic proteins Bcl‐2 and Bcl‐xL promoting cell survival.^131^ However, PKA activity had also been linked to apoptosis through the phosphorylation and stabilization of Bim on Ser83,^130^ and PKA phosphorylation of Bax on Ser60 promotes Bax translocation to mitochondria and MOMP.^132^ As with protein import, cell context and the dose/duration of signaling should be considered when evaluating local outcomes of mitochondrial PKA signaling.

Given the reliance of mitochondria on local PKA signaling, it is not surprising to find that perturbations in PKA signaling can be found in neurodegenerative disorders typified by mitochondrial dysfunction.^133^, ^134^, ^135^, ^136^ In preclinical models of PD, there is diminished PKA signaling that coincides with data collected in postmortem brains. Increasing the levels of mitochondrial PKA either through increasing AKAP‐1, targeting PKA to mitochondria, or pharmacological activation of PKA can offset mitochondrial dysfunction and neuronal cell death in PD models, in particular, those with PINK1 deficiency.^96^, ^97^, ^99^, ^104^ Similar to PD, dysregulation of PKA signaling has been noted in AD and models with elevated amyloid beta.^93^ Defects in mitochondrial health and trafficking in AD‐like conditions were alleviated by the reintroduction of PKA.^93^, ^135^ These results indicate that diminished PKA signaling is a component of neurodegenerative disorders and restoring local PKA activity could be a means to impede the pathogenesis of these conditions.

### 4.2 Protein kinase C (PKC)

Protein kinase C isoforms are emerging as potential regulators of mitochondrial function in the areas of metabolism and apoptosis^137^, ^138^, ^139^; however, a complicating factor could be that distinct activities of PKC isoform could have discrete impacts on mitochondrial physiology. To date, three isoforms of PKC, PKCα,^140^ PKCδ, and PKCε,^141^ have shown mitochondrial localization. PKCδ is associated more with neurodegeneration and neuroinflammation,^142^, ^143^ while PKCε has been demonstrated to be more protective.^144^

PKCδ has been shown to undergo cleavage by caspase activity in response to stress, and the cleaved species of PKCδ contributes to stress‐induced mitochondrial dysfunction. PKCδ‐induced mitochondrial dysfunction is also associated with dopaminergic neurotoxicity in preclinical PD models.^143^, ^145^ Although the outcomes of mitochondrial PKCδ signaling affect apoptosis, respiration, membrane potential, ROS production, antioxidant capacity, and organelle dynamics,^146^, ^147^, ^148^ only Drp‐1 has been shown to interact with PKCδ and contribute to organelle fragmentation.^149^ Further investigation is needed to elaborate on PKCδ functions at mitochondria.

PKCε also demonstrates mitochondrial localization in response to cerebral ischemia and increases sirtuin 5 (Sirt5) levels.^150^ Outside of this report, most of the work has been performed in non‐CNS tissues. PKCε has been shown to phosphorylate complex IV and increase its activity in the heart and kidney in response to stress.^138^, ^151^ Also, PKCε has been shown to phosphorylate and regulate cardiac sodium channels in response to metabolic changes. Concerning the CNS, PKCε phosphorylation has been shown to increase the activity of endothelin converting enzyme (ECE), which is responsible for amyloid beta clearance, via an N‐terminal modification site.^152^ This would indicate that PKCε may be downregulated in Alzheimer's disease,^153^ and restoring PKCε activity could be neuroprotective similar to how PKCε activity is observed in cardiac tissue.

## 5 PARKINSON'S DISEASE RELEVANT PROTEIN KINASES

**Figure 4:**
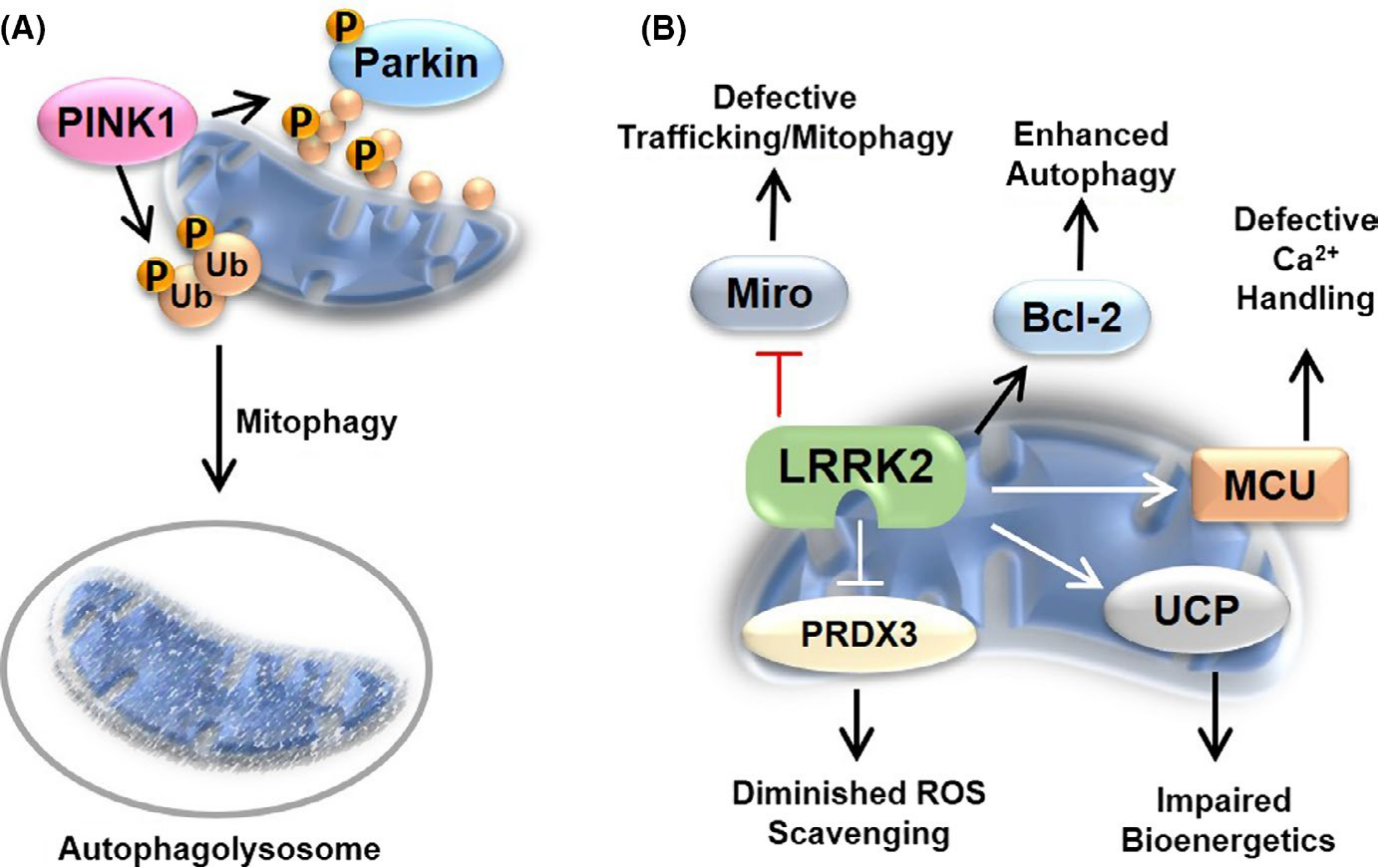
Parkinson's disease‐related protein kinases impact mitochondrial physiology. PD is associated with mitochondrial dysfunction, and numerous proteins implicated in familial PD or disease pathogenesis exhibit mitochondrial localization or influence organelle physiology. (A) Loss‐of‐function mutations in PINK1 are found in familial PD patients. PINK1 accumulates at the OMM following mitochondrial depolarization leading to its activation by autophosphorylation. An activated PINK1 can then phosphorylation ubiquitin on Ser65 (Ub—orange circles) and activate/recruit Parkin to stress, aged, or damaged mitochondrial to facilitate their elimination by mitophagy. (B) Mutations in LRRK2 increase kinase activity and occur in both familial and sporadic PD cases. Increased LRRK2 activity can be linked to changes in autophagic flux through the manipulation of Bcl‐2 and inhibition of Miro, whereby LRRK2 can interact with and sequester Miro preventing turnover and trafficking of damaged organelles. LRRK2 mutants, such as G2019S, are also associated with changes in Ca^2+^ homeostasis through perturbation of mitochondrial calcium uniporter (MCU) and decreased oxidative stress tolerance by phosphorylation‐dependent impairment of PRDX3. LRRK2 variants are found to affect bioenergetics by inhibiting complex I (not shown) and increasing UCP abundance and stability. The kinases represent direct effectors of PD‐induced mitochondrial dysfunction

Protein kinases have been implicated in the pathogenesis of Parkinson's disease (PD), and discrete changes in protein kinase activity have been noted for cytosolic kinases like PKA and JNK that promoted disease physiology (described above). However, two kinases in particular are linked to familial and idiopathic cases of PD and have populations present on the OMM. Mutants of the leucine‐rich repeat kinase 2 (LRRK2) and the PTEN‐inducible kinase 1 (PINK1) can profoundly impact organelle physiology contributing to PD pathogenesis and progression. (summarized in Figure 4).

### 5.1 Leucine‐rich repeat kinase 2 (LRRK2)

A protein kinase implicated in familial and sporadic cases of PD is leucine‐rich repeat kinase 2 (LRRK2), wherein mutations in LRRK2 have been shown to increase kinase activity.^154^ LRRK2 and mutant LRRK2 localize to intracellular membranes in the rodent brain and human cells. Intriguingly, approximately 10% of LRRK2 has been shown to localize to the OMM,^155^ and there is increasing evidence for mutant LRRK2 to directly impact mitochondrial physiology and apoptotic machinery (reviewed in 156). Also, mutant LRRK2‐induced mitochondrial dysfunction has been noted in numerous experimental models and PD patients.

Increased LRRK2 kinase activity has been linked to the phosphorylation of and interactions with mitochondrial proteins implicated in organelle dysfunction in PD^157^ including the uncoupling proteins in neurons,^158^ Bcl‐2 proteins,^159^ Drp‐1,^160^, ^161^, ^162^, ^163^ Miro,^164^ the mitochondrial calcium uniporter (MCU),^165^ and peroxiredoxin 3 (PRDX3).^166^ Early studies found that expression of the most common clinical LRRK2 mutant G2019S could impact mitochondrial morphology increasing mitochondrial fission. The enhanced fission was found to be a result of interaction between LRRK2:G2019S and Drp‐1.^160^ The recruitment of Drp‐1 to mitochondrial and subsequent fission in PD patient‐derived fibroblasts and human SH‐SY5Y cells was found to be dependent upon LRRK2 kinase activity.^161^, ^162^ Given the recent identification of Rab GTPases, specifically Rab7,^167^ as bonafide LRRK2 substrates,^168^ it is likely that mutant LRRK2 activity could drive mitochondrial fragmentation observed in PD patients by assembling the protein machinery and activating membrane fusion.^169^

LRRK2: G2019S expression in HeLa cells revealed that LRRK2 mutants interacted with and phosphorylated Bcl‐2 on Thr56 to increase autophagy and mitochondrial mass.^159^ However, Hsieh and colleagues reported that LRRK2 in iPSC‐derived neurons facilitate the interaction of damaged mitochondria with the microtubule motor accessory protein Miro paving the way for clearance of depolarized organelles; consequently, LRRK2:G2019S impedes the interaction of Miro with damaged mitochondria slowing the clearance of potentially detrimental organelles from neurons.^164^ Additionally, LRRK2:G2019S is linked to aberrant mitochondrial trafficking, specifically axonal transport, through inhibition of the Miro‐mitochondrial interaction.^164^ Fibroblasts from PD patients do exhibit more mitochondrial fragmentation and enhanced mitophagy initially compared to control cells. Thus, the role of LRRK2 in mitophagy will require a closer examination.^169^

Lastly, enhanced LRRK2 kinase activity can affect ROS levels and production through manipulation of mitochondrial substrates. While LRRK2 mutants are known to alter ETC function, LRRK2 mutants have been found to alter antioxidant capabilities in mitochondria. For example, LRRK2:G2019S was found to phosphorylate and impair PRDX3 in drosophila brains diminishing organelle oxidant handling capacity.^166^ Alternatively, patient‐derived fibroblasts and SH‐SY5Y cells expressing LRRK2:G2019 have elevated levels of uncoupling protein expression, namely UCP2 and UCP4, respectively.^158^ Further, the increased expression of UCPs linked to kinase activity, as the elevation in UCP mRNA was reversed by LRRK2 inhibitors. The increase in UCP appears to be neuroprotective because UCP inhibitors exacerbate LRRK2 mutant pathology^158^ and overexpression of UCP2 protects against PD‐relevant neurotoxins, such as MPTP.^158^ Thus, UCPs may protect by diminishing the production of ROS by aberrant ETC function and compensate for diminished organelle antioxidant capacity. A recent study demonstrates that mutant LRRK2 can influence the MCU increasing intraorganellar Ca^2+^ concentrations.^165^ This elevation in Ca^2+^ may induce ROS production in LRRK2 mutant cells. These studies collectively demonstrate that LRRK2 is a crucial component of mitochondrial regulation.

### 5.2 PTEN‐induced kinase 1 (PINK1)

PTEN‐induced putative kinase 1 (PINK1) has drawn significant attention due to its role in mitophagy and association with PD. Under basal conditions, PINK1 is transported into the mitochondrial matrix through the translocases of the outer membrane, TOM20 and TOM23, the translocation channel TOM40, and the translocase of inner membrane TIM23. Inside the mitochondrial matrix, PINK1 is cleaved by a mitochondrial processing peptidase (MPP) and a rhomboid protease, PARL.^170^, ^171^, ^172^ The cleaved PINK1 is then re‐transported into the cytosol and degraded by the ubiquitin (Ub) proteasome system (UPS).^171^

When PINK1 transport through TOM is hindered, for instance by a depolarized mitochondria membrane potential (MMP), it accumulates on the OMM and auto‐phosphorylates at Ser228 and Ser402.^173^, ^174^ The activated PINK1 phosphorylates Ub chains at Ser65,^175^, ^176^ which recruit Parkin to the OMM^177^ and activate Parkin's E3 Ub ligase activity via phosphorylation.^178^ Just as PINK1 phosphorylates Ub, PINK1 also phosphorylates Parkin directly at Ser65,^179^ signifying a secondary mode of Parkin activation.^180^, ^181^ The phosphorylated Parkin translocates to the mitochondria and is further activated by the PINK1‐phosphorylated Ub.^177^ Parkin's subsequent ubiquitination of OMM substrates leads to an enrichment of Ub molecules around the damaged mitochondria that enter a positive feedback cycle through PINK1 phosphorylation.^182^

PINK1 can also alter OMM signal transduction to drive fission. Activation of PINK1 destabilizes the interaction between PKA and anchoring protein AKAP‐1, preventing unnecessary mitophagy.^178^ The dissociation of PKA from AKAP‐1 prevents phosphorylation and inhibition of Drp‐1 leading to increased fission. Alternatively, PKA can translocate intracellularly and phosphorylates the mitochondrial membrane protein MIC60 on Ser528, preventing PINK1 accumulation on the OMM and reducing Parkin recruitment to mitochondria.^105^ In the presence of mitochondrial pathology, PINK1 likely drives reduced mito‐PKA and AKAP, allowing PINK1 to flag the damaged mitochondria for degradation through this feedback mechanism.

Accumulation of mitochondrial mutations is observed in the *substantia nigra* dopaminergic (DA) neurons of PD patients that, due to their high metabolic needs, may result in neuronal death. As most mutations associated with PINK1 are in the kinase domain,^183^, ^184^ the adenosine triphosphate (ATP) analogy kinetin triphosphate (KTP) can be used to interact with PINK1, allowing for kinase activity and Parkin‐induced mitophagy independent of the mutated kinase domain.^185^ Huntington's disease (HD) can be characterized by the presence of ring‐shaped neuronal mitochondria which are formed in the presence of reduced mitophagy.^186^ Overexpressing PINK1 in HD models reduces the formation of the spheroid mitochondria, decreases the number of damaged mitochondrial, and counteracts neurotoxicity.^187^ In Alzheimer's disease (AD), ectopic expression of PINK1 may be capable of reducing or even preventing, the amyloid‐β (Aβ) plaques that characterize the neurological disease as an accumulation of dysfunctional mitochondria and oxidative damage have been shown to precede the Aβ in AD.^188^ Thus, diminished PINK1 activity could be a basal component of neurodegenerative disease pathogeneses.

## 6 OTHER KINASES ASSOCIATED WITH OMM

As mentioned earlier, the occurrences of cytosolic protein kinases are increasing and more OMM kinases are being found through disease‐specific proteomic studies. These include protein kinases that are implicated in cellular homeostasis, such as AMP‐dependent protein kinase (AMPK), casein kinases (CK1 and CK2), and mammalian target of rapamycin (mTOR). Intriguingly, kinases implicated in the cell cycle have also emerged as OMM signaling agents. The appearance of these kinases on the OMM is further evidence of the extensive integration of organelle and cell physiologies and quality control.

### 6.1 AMP‐dependent protein kinase (AMPK)

A recent addition to the OMM signaling landscape is the AMP‐dependent protein kinase (AMPK), which was identified in two independent reports.^120^, ^189^ First, Toyama and colleagues 189 demonstrated that AMPK's response to ETC stress (rotenone or antimycin treatment) leads to the activation of AMPK and ultimately the colocalization with TOMM20. This study also revealed that mitochondrial fission factor (MFF) was phosphorylated by AMPK on Ser172, which was required along with Ser155 phosphorylation for the recruitment of Drp1 and ultimately stress‐induced mitochondrial fragmentation.^189^ Moreover, phosphorylation of Mff on Ser172 and Ser155 was found to be essential to the proper distribution of dendritic mitochondria in mouse primary cortical neurons.^189^ This study was important because it placed AMPK in proximity to ATP production and provided a mechanistic link between mitochondrial bioenergetics and dynamics.

Alternatively, Hoffman et al^120^ utilized phosphoproteomics to identify substrates of AMPK in continuous cell cultures and muscles. One of the validated substrates was an OMM scaffold for PKA, AKAP‐1. AMPK phosphorylation of AKAP‐1 on Ser103 leads to increased respiration in rat L6 myoblasts likely in a PKA‐dependent manner.^120^ As mentioned earlier, enhanced mitochondrial PKA activity is linked to the improved import of ETC components and phosphorylation of ETC enzymes by PKA improves activity. Therefore, AMPK activity on the OMM is a means to facilitate ETC activity by sustaining PKA activity through manipulation of the scaffold AKAP‐1. These studies demonstrate that AMPK is an essential integrator of bioenergetic status^190^, ^191^ and organelle dynamics^192^ on the mitochondrial surface, and AMPK activity on mitochondria may be a player in CNS disorders such as PD^193^and ALS.^194^

### 6.2 Cyclin‐dependent kinase 1 (Cdk1)

Cyclin‐dependent kinase 1 (Cdk1) complexed with cyclin B is known to impact mitochondrial substrates during mitosis. Cdk1 was first reported to impact mitochondrial proteins in 2007 when Taguchi and colleagues discovered that Cdk1/cyclin B could phosphorylate Drp‐1 on Ser585 (Ser616 human) and promoting mitochondrial fission prior to cell division.^195^ It was later demonstrated that Cdk1/cyclin B could impact matrix proteins as well including components of respiratory complex I. The phospho‐manipulation of the complex I subunits ultimately promoted the respiration necessary to produce the bioenergetic currency necessary for cell division.^196^ Both the regulation of mitochondrial fission and respiratory chain by Cdk1/cyclin B have been linked to oncogenesis and therapeutic resistance in brain tumors as well as other types of cancer.^197^ Collectively, mitochondrial signaling by Cdk1/cyclin B is an excellent example of the extensive coordination between mitochondrial and cellular programming.

### 6.3 Cyclin‐dependent kinase 5 (Cdk5)

Another example of a Cdk exhibiting OMM signaling capabilities is cyclin‐dependent kinase 5 (Cdk5). Like Cdk1, Cdk5 can phosphorylate Drp1 on Ser616 in mature rat neurons, the same site as Cdk1 in mitotic cells, inhibiting mitochondrial fission.^198^ Cdk5 activity was associated with elongated mitochondria during the maturation of cultured cortical neurons.^198^ Intriguingly, Cdk5‐mediated phosphorylation of Drp1 is linked to oncogenic potential in brain tumor initiating cells, in which enhanced Drp1 activity correlated with a poor glioblastoma prognosis.^199^ In another study, Cdk5 was crucial to N‐methyl‐D‐aspartate (NMDA)‐induced neuron loss, and elimination of Drp1 phosphorylation or the catalytic activity of Cdk5 was sufficient to prevent NMDA‐induced cell death.^200^ Furthermore, cerebral granule cells derived from *Cdk5^‐/‐^* mice were resistant to NMDA‐induced cytotoxicity compared to controls.^200^ Cdk5 phosphorylation of Drp1 also occurs in models of neurodegenerative disorders. Cdk5‐mediated phosphorylation of Drp1 can promote fission and apoptosis in striatal neurons that express mutant Huntington, an event that can be exacerbated by the addition of dopamine.^201^ This study implicates Cdk5‐Drp1 activation as a component of Huntington disease. Additionally, Cdk5 phosphorylation of Drp1 occurs in mouse primary cortical neurons exposed to amyloid beta (Aβ_1‐42_).^202^ Neurons treated with Aβ_1‐42 _exhibited increased fission and apoptosis that could be ablated by impairing Cdk5, which may indicate that Cdk5 activation of Drp1 may be involved in AD pathophysiology.^202^ Intriguingly, these reports place a cell cycle enzyme in stress response pathways within postmitotic cells.

### 6.4 Cyclin‐dependent kinase 11 (Cdk11)

Similar to Cdk5, cyclin‐dependent kinase 11 (Cdk11) has been shown by colocalization studies to have subpopulation on mitochondria.^203^, ^204^ After the identification of Cdk11 on mitochondria, there have been no follow‐up studies to identify substrates. Intriguing the localization of Cdk11 preceded the induction of apoptosis leading investigators to surmise that Cdk11 may have a role in the induction of cell death.^203^, ^204^ Cdk11 represents an interesting OMM kinase for study, as research related to mitochondria. Cdk11 could shed light on the relationship between organelle physiology and the cell cycle.

### 6.5 Casein kinase I (CK1)

Both casein kinase I and II (CK1 and CK2) have been described to have mitochondrial influence and localization.^124^, ^205^, ^206^ CK1 and CK2 are commonly viewed as Ser/Thr kinases that influence the activity of transcription factors to influence developmental signaling (ie, Wnt pathway) and circadian rhythms. However, CK1 and CK2 have been implicated in the regulation of mitochondrial function. CK1 is involved the inhibition of Fas‐mediated apoptosis through the phosphorylation of Bid on Ser64 and Ser66.^207^, ^208^ Phosphorylation by CK1 prevents the cleavage of Bid by caspase 8 limiting the release of cytochrome c from mitochondria.^207^, ^208^ While specific CK1 targets on mitochondria have yet to be identified, CK1 has been colocalized with mitochondria, and further studies may unearth CK1 substrates on mitochondria.

### 6.6 Casein kinase II (CK2)

The role of CK2 on mitochondria is more extensive than CK1 where in addition to apoptosis CK2 plays a significant role in the regulation of organelle protein import^122^, ^123^ and quality control.^206^ Concerning apoptosis, CK2 is known to phosphorylate Bcl‐2 proteins and affect their activities. An example includes, like CK1, CK2 can phosphorylate Bid preventing the induction of apoptosis, activation of caspases, and sustaining the levels mitochondrial Bcl‐2.^207^ Recent data suggest CK2 has a more prominent role in the regulation of mitochondrial protein import. CK2 has been associated with the phosphorylation of OMM protein transport components TOM22 and TOM70.^122^, ^123^, ^124^, ^125^ In both cases, CK2 enhances the localization and function of these transport proteins in the OMM and thus positively impacts protein transit into mitochondria. Additionally, CK2 phosphorylation of Mim1 complex stabilizes the protein in the OMM and leads to increased activity including the incorporation of TOM22 into the OMM and TOM super‐complex.^122^, ^123^, ^124^, ^125^ CK2 is implicated in mitophagy as well.^206^, ^209^, ^210^ CK2 phosphorylation of OMM mitophagy receptor FUN14 domain containing 1 (FUNDC1)^211^ on Ser13 prevents the interaction of mitochondria with LC3 reducing mitophagy perhaps as a means to retain healthy mitochondria.^210^ Therefore, CK2 has emerged as a crucial element of mitochondrial physiology and defects in CK2 function could adversely affect organelle physiology and related human conditions.

### 6.7 Mammalian target of rapamycin (mTOR)

The mTOR signaling pathway has long been implicated in sensing mitochondrial substrates like branched amino acids; moreover, mTOR has been linked to bioenergetics and mitochondrial protein synthesis for some time.^212^, ^213^ The localization of mTOR signaling components on lysosomes and in proximity to mitochondrial‐associated membranes (MAMs) meant it was only a matter of time before mitochondrial mTOR substrates would be discovered.^214^ Indeed, mTOR was reported to localize to mitochondria via the OMM protein FKBP38, a member of the FK506‐binding protein family.^215^ This interaction places mTOR in close to Bcl‐2 proteins associated with VDAC1.^213^, ^216^ Bcl‐2 proteins associated with VDAC1 influence substrate entry into mitochondria and dictate the cellular reliance on glycolysis. A recent report demonstrates that mitochondrial mTOR can phosphorylate Bcl‐xL on Ser62 and this increased cellular reliance on glycolysis in leukemia cells.^213^ The localization of mTOR places the kinase near other regulators including AMPK making mTOR an emerging regulator of mitochondria function capable of responding to changes in redox status and bioenergetics shifts. Alternatively, mTOR may have a role in OMM signaling through interplay with AKAP‐1, wherein mitochondrial PKA signaling may influence mTOR activity.^217^ It is clear that mTOR signaling is highly integrated with mitochondria physiology.^212^, ^218^, ^219^

### 6.8 P21‐activated kinase 5 (PAK5)

The p21‐activated kinase 5 (PAK5) is a Ser/Thr protein kinase that primarily regulates cytoskeleton dynamics and contributes to cell proliferation.^220^ PAK5 exhibits Rho‐kinase‐dependent mitochondrial localization.^221^, ^222^ PAK5 was shown to phosphorylate Bad on Ser112 and prevent Bad migration to mitochondria.^223^ This increases levels of Bcl‐2 and prevents apoptosis.^223^ Further investigation is warranted to determine whether PAK5 can phosphorylate other BH3‐only proteins and whether these modifications coincide with changes in cytoskeletal dynamics.

The number of reported protein kinases localizing to mitochondria continues to grow at an increasing pace. The reports of new signaling kinases on the OMM reflect the structural and functional integration of mitochondria with other subcellular compartments. Thus, the kinases presented above may only be a modest sampling of the cascades present on the mitochondrial surface.

## 7 OMM PROTEIN PHOSPHATASES

Compared to the breadth of studies on OMM protein kinases, far less is known regarding the activities and substrates of protein phosphatases on the mitochondrial surface. Because a number of the phosphorylation events ascribed to protein kinases on the OMM are reversible, it is likely that some phosphatases would be required to offset and regulation these phosphorylation events to adjust and maintain organelle physiology.^8^, ^11^ We will describe the actions of some of the known protein phosphatases on mitochondria below.

### 7.1 Mitogen‐activated protein kinase phosphatase‐1 (MKP‐1)

MKP‐1 is a dual specificity phosphatase capable of dephosphorylating Ser/Thr and Tyr residues modified by protein kinases.^224^ As the name implies, the primary substrates of MKP‐1 are the MAPKs. As described earlier, MAPKs (namely JNK, ERK1/2, and p38) can translocate to mitochondria and manipulate local proteins. MKP‐1 was also found to migrate to neuronal mitochondria in response to treatment of cells with neuronal growth factor.^224^, ^225^ It is proposed that JNK is a substrate of MKP‐1 on mitochondria based on brain‐related studies 226, 227, 228 suggesting that MKP‐1 may act to inactivation JNK signaling to prevent apoptosis. Further investigation is warranted to determine whether the phosphatase affects other MAPKs on mitochondria or has unique substrates on the OMM.

### 7.2 Phosphate and tensin homolog‐long (PTEN‐L)

A recent report by Wang et al introduced the presence of the long PTEN isoform, PTEN‐L, on the OMM.^229^ Ectopic expression of PTEN‐L impaired mitophagy by preventing Parkin translocation and reducing the levels of phosphorylated ubiquitin on the mitochondrial surface. Ablation of PTEN‐L expression promoted mitophagy suggesting that PTEN‐L could be a negative regulator of PINK1‐dependent mitophagy.^229^ Intriguingly, site‐directed mutagenesis to impair the lipid phosphatase activity of PTEN‐L did not impact its role in the control of mitophagy.^229^ This study indicates that PTEN‐L has a unique conformation that permits protein phosphatase activity on the OMM.

### 7.3 Protein phosphatase 1 (PP1)

The PP1 is linked to the regulation of local signaling transduction on the OMM and the function of proteins related to apoptosis.^230^, ^231^, ^232^, ^233^ PP1 forms a complex with PKA and AKAP1 on the mitochondrial surface,^234^ and in the absence of PKA, PP1 can destabilize AKAP1 leading to its degradation and reduce potential sites for mitochondrial PKA signaling.^235^ This event was linked to the induction of long‐term depression through the dephosphorylation of AMPA receptor.^234^ OMM PP1 has been cited as a regulator of p38 signaling on mitochondria as well. PKC was found to induce PP1 following exposure to phorbol ester leading to dephosphorylation and inactivation of p38 on the mitochondria.^231^ These studies indicate that PP1 could modulate OMM signaling in a context‐dependent fashion.

PP1 is associated with the dephosphorylation of Bcl‐2 proteins and the induction of apoptosis.^232^ PP1 dephosphorylation of Bad can release the protein from 14‐3‐3 and permits Bad's interaction with and inhibition of anti‐apoptotic Bcl‐2 proteins.^128^ PP1 interaction with Bcl‐2 and Bcl‐xL can also impact phosphatase activity.^236^ These results indicate PP1 on the OMM is a regulator of apoptotic proteins.

### 7.4 Protein phosphatase 2A (PP2A)

Similar to PP1, PP2A has been shown to have mitochondrial localization to the OMM, where it can influence the activities of Bcl‐2 proteins and Bad by dephosphorylation.^237^, ^238^ However, a distinction between PP1 and PP2A substrates appears to be the ability of PP2A to dephosphorylate Drp‐1 and activate the protein. PP2A was shown to modify Ser656, the same residue phosphorylated by PKA, and undo PKA‐mediated inhibition of Drp‐1 leading to mitochondrial fragmentation and depolarization.^95^ In neurons, PP2A activation of Drp‐1 led to decrease synapse formation and shorter and fewer dendrites.^95^ Excessive PP2A activity could be a player in neurological conditions that may have fragmented mitochondrial networks, such as PD.

### 7.5 Protein tyrosine phosphatase, non‐receptor type 11 (PTP1D)

PTP1D is another phosphatase that has been associated with AKAP1 on the OMM.^239^ PTP1D in response to EGF signaling can dephosphorylate Src activating its kinase activity. Increased Src activity is associated with increased ETC activity by direct phosphorylation of subunits by Src.^239^ In such, PTP1D may act to supplement PKA signaling through a shared molecular interaction with AKAP1.

Given the diversity of signaling molecules present on the mitochondrial surface and the multitude of physical and functional interfaces of the OMM, it is likely that the number of protein phosphatases will increase to regulate the growing populations of protein kinases on the cell surface. Further investigation into the dynamic assembly and disassembly of OMM signaling cascades may reveal the identity of new OMM phosphatases or new substrates for existing OMM phosphatases.

## 8 OMM SCAFFOLD PROTEINS

Scaffold proteins and adaptor proteins are crucial accessory proteins required for the coordination of signal transduction events across space and time.^240^, ^241^ The relative abundance of accessory proteins can dictate the biological outcomes of signaling events by influencing the spatial magnitude and duration of a response.^240^, ^241^ Therefore, the identification and concentrations of these elements of molecular architecture will be critical to determine the presence and impact of signaling pathways on mitochondria.

### 8.1 A‐kinase anchoring protein 1 (AKAP‐1)

AKAPs are membrane‐bound scaffolds for PKA and its related signaling components.^91^ AKAP‐1 can exhibit mitochondrial localization (often referred to as D‐AKAP1), and reports have placed it on and within mitochondria.^242^ Of note, the mitochondrial variants of the *Akap1* gene most often associated with the OMM are sAKAP‐84, AKAP‐121, and AKAP‐149.^242^ Single nucleotide polymorphisms in *Akap1* can alter the subcellular distribution of AKAP1 variants and alter the outcomes of cAMP/PKA signaling.^243^ Indeed, the relative levels of AKAP1 on the mitochondria can influence the outcomes of local PKA signaling.^95^, ^96^, ^244^, ^245^, ^246^, ^247^ By merely increasing AKAP‐1 concentrations on mitochondria, investigators have been able to leverage the prominent concentrations of PKA in the cytosol to influence mitochondrial health. Overexpression of AKAP‐1 can protect neurons and neuron‐like cells against PD relevant neurotoxins and PINK1 deficiency as noted earlier. Furthermore, genetic ablation of AKAP1 was found to exacerbate stroke‐related injury in mice.^100^ The deletion of *Akap1* in mice reduced phosphorylation of Drp‐1 on Ser637, which impaired fission.^100^ Reduced Drp1 phosphorylation in *Akap1^‐/‐^* mice contributed to Ca^2+^ dysregulation, dysregulation of complex II, and increased ROS production in response to excitotoxic stress.^100^ This suggests that the PKA/AKAP1 mitochondrial signaling nexus is crucial to regulating stress responses and preserving optimal organelle function in the brain.

A recent report indicates that mitochondria AKAPs are arranged into specialized membrane microdomains and this physical interaction places PKA proximal to mitochondrial substrates such as Bad.^248^ These microdomain associations are greatly influenced by local phosphatases capable of influencing the stability of PKA signaling on the OMM. Recently, post‐translational modification of AKAP‐1 has been shown to affect protein stability on the OMM as well. Dephosphorylation of AKAP‐1 can lead to its ubiquitination by E3‐ubiquitin ligase seven in absentia 2 (Siah2) resulting in its degradation.^244^ This ultimately diminishes PKA concentrations on mitochondria, which can impair the beneficial effects of local PKA signaling.^244^ Given the anomalies related to PKA signaling in neurodegenerative disorders, it is likely that disorientation and destabilization of AKAP‐1 on the OMM could be responsible for the lack of mitochondrial health in conditions such as AD and PD. Restoring optimal levels of mitochondrial PKA signaling could help restore organelle function and reduce the detrimental effects of mitochondrial perturbations in neurodegenerative disease.

### 8.2 Sab (SH3‐binding protein 5; SH3BP5)

As mentioned above, Sab is an OMM scaffold for the JNK and a putative substrate of p38γ on the OMM.^27^, ^28^ To date, no other outer membrane kinases have been shown to interact with Sab. This could be due to the topology of Sab, which may make it selective for MAPKs on the OMM. Near the C terminus of Sab, there are two kinase interacting motifs (KIM1 and KIM2) (see Figure 2). The Sab KIM1 motif is immediately followed by a transmembrane motif, which would indicate that KIM2 is the only kinase interacting motif on the cytosolic side of the OMM. Previous studies have indicated that JNK interacts with Sab through the KIM1 motif, while the KIM2 motif was dispensable for JNK‐mediated phosphorylation.^29^, ^55^ A study by Win et al^29^ demonstrating the topology of Sab indicates that JNK is on the cytosolic face of the OMM and likely interacting with KIM2 of Sab. A recent study from our laboratory indicates that JNK phosphorylates Sab on Ser421 which is near KIM2 indicating that KIM2 is likely the binding site for JNK on the OMM.^32^ Selective inhibition of the JNK‐Sab interaction reveals that KIMs are necessary for JNK signaling on the mitochondria and JNK‐related mitochondrial activities.^26^, ^31^, ^32^, ^33^, ^54^, ^249^, ^250^ The concentrations of Sab on the OMM dictate the outcomes of mitochondrial JNK signaling.^31^, ^32^, ^250^ Recent studies from our laboratory demonstrate that increasing Sab levels in mitochondria renders cells vulnerable to stresses due to elevated levels of JNK signaling on the OMM. Demonstrating that the relative levels on scaffold proteins on the OMM dictate signaling outcomes, and the collective levels of distinct scaffolds could indicate how mitochondria may respond to cellular cues and stresses. We have found that Sab mRNA and protein levels are significantly enriched in the hippocampus, substantia nigra, and cerebellum, areas of the brain vulnerable to neurotoxin stimuli.^251^ Thus, we contend that the elevated Sab concentrations could lead to robust mitochondrial JNK activity contributing to apoptosis and neurodegeneration in disorders such as AD and PD. Selective disruption of the JNK‐Sab interaction protects dopaminergic neurons from 6‐hydroxydopamine‐induced neurotoxicity.^33^ Thus, the specific targeting of detrimental mitochondrial JNK signaling could be a promising target to preserve mitochondrial health in PD.

### 8.3 Protein interacting with C‐kinase‐1 (PICK1)

PICK1 is a scaffold protein for PKCs, and PICK1 has been linked to PKAα localization on mitochondria, where the kinase protected against apoptosis induced by genotoxic stress.^252^, ^253^, ^254^ Another report indicates that PICK1 on mitochondria can impair Parkin activity and contribute to MPTP‐induced neurotoxicity. PICK1 destabilized the interaction between Parkin and UbcH7 leading to diminished E3 ubiquitin ligase activity of Parkin. PICK1 knockout mice were protected against MPTP‐induced cell death.^255^ This effect of PICK1 is interesting in light of the neurotoxic and neuroinflammatory activities of PKCδ and may be another mechanism by which shifts in OMM signaling can contribute to PD pathogenesis and progression.^256^

Additional scaffold proteins may also be localized to the OMM, as reports indicate that 14‐3‐3 may have a population of mitochondrial proteins that influence organelle signaling.^257^, ^258^ Also, the growth factor receptor‐bound protein 10 (Grb10) may have OMM localization.^259^ We contend that the accurate and reliable identification of OMM scaffold proteins will be crucial to determining the localization and signaling pathways to the mitochondrial surface.

## 9 CONCLUDING REMARKS AND FUTURE DIRECTIONS

In this review, we have presented some of the emerging signaling cascades and phosphorylation events on the mitochondria surface. OMM signaling components are appropriately positioned to impact specific aspects of mitochondrial physiology. It is likely that more rigorous studies in this area will unearth more kinases, phosphatases, and adaptor proteins. Great care should be taken in identification and validation of new signaling components on the OMM using established as well as modern proteomic‐based approaches.

While the identification of OMM signaling components and signaling cascades continues to be of interest, many of the OMM kinases are beginning to demonstrate interplay on the organelle surface. Earlier, we mentioned that AMPK could stabilize PKA signaling on mitochondria to enhance ETC function.^120^ However, antagonism by kinases may also occur. The best current evidence for this is that PINK1 can impair the kinase activity of LRRK2,^260^, ^261^, ^262^ an effect which is lost in iPSC‐derived neurons from PD patients harboring PINK1 mutations. Similarly, overexpression of Parkin can prevent dopaminergic neuron loss in the presence of LRRK2:G2019S.^260^, ^261^ In addition to modulating protein function, it is likely that signaling cascades can target opposing pathways for degradation using by manipulating local E3‐ubiquitin ligases or other turnover mechanisms (ie, autophagy). But, one can anticipate that the scaffolding and organization of these molecular pathways will be critical to this integration. This potential has been noted with AKAP1, which plays a crucial role in the integration of PKA and mTOR signaling modules.^217^ It is likely that similar mechanisms exist for other signaling proteins to balance mitochondrial form and function in an everchanging cellular environment. Research oriented around these distinct interactions will be critical to understanding the complex regulation of mitochondrial function and dysfunction in disease pathogenesis, and this scenario also highlights the need to consider therapeutic regimens that impair detrimental events while restoring beneficial signaling pathways on the OMM.

It is likely that the OMM signaling pathways will be highly responsive to mitochondrial messengers, such as ROS, and OMM proteins are appropriately positioned to recognize and response to these small molecules. The response to these messengers will shape cellular responses to the organelle health status. An example of this type of integration may be best illustrated by the interplay between ROS and the members of the Bcl‐2 superfamily that dictate apoptotic responses.^263^ For example, Bcl‐2 can “sense” the magnitude of ROS production in response to post‐translational modifications, namely Ser70 phosphorylation,^264^ and changes in Bcl‐2 can then impact ETC function, mitochondrial antioxidant capacity, and autophagic flux.^263^ The robustness of the Bcl‐2 response to ROS levels can determine whether a cell undergoes apoptosis. Our work demonstrates that enhancing mitochondrial JNK signaling by increasing Sab levels amplifies ROS production and renders cells sensitive to chemical insults in part by diminishing OMM Bcl‐2 levels.^31^, ^32^, ^250^ It is established that mitochondrial ROS can trigger ASK‐1 to engage sustained JNK activity^265^ leading to depletion of OMM Bcl‐2 by Ser70 phosphorylation and induction of apoptosis.^26^ In addition to JNK, numerous OMM localized kinases and phosphatases can modulate Bcl‐2 superfamily protein function, which we contend is one significant route to coordinating organelle and cellular physiology because of the importance of Bcl‐2 proteins to metabolism, autophagy, and apoptosis. The actions of OMM signaling proteins in response to ROS could dictate the actions of Bcl‐2 proteins within the cell. Thus, it is imperative that OMM signaling pathways converging on Bcl‐2 proteins be considered with great respect toward cellular environment.

In conclusion, the signalosomes of the OMM are the gatekeepers of mitochondrial physiology and mediators of disease pathogenesis in the CNS, as such there is much promise in the elucidation of signaling relationships on the mitochondrial surface and targeting distinct OMM signaling components to combat CNS disorders.

## CONFLICT OF INTEREST

The authors declare no conflict of interest.
